# Adult Movement Pattern and Habitat Preferences of the Maghribian Endemic *Gomphus lucasii* (Odonata: Gomphidae)

**DOI:** 10.1093/jisesa/iev128

**Published:** 2015-10-28

**Authors:** Rabah Zebsa, Rassim Khelifa, Amin Kahalerras

**Affiliations:** ^1^Département d’écologie et du génie de l’environnement, Faculté des Sciences de la Nature et de la Vie et des Sciences de la Terre et de l'Univers, Université 08 Mai 1945, Guelma 24000, Algérie; ^2^Institute of Evolutionary Biology and Environmental Studies, University of Zurich, Winterthurerstrasse 190, CH-8057 Zurich, Switzerland; ^3^Département de biologie, Faculté des Sciences biologiques et agronomiques, Université de Tizi Ouzou, Tizi Ouzou 15000, Algérie

**Keywords:** *Gomphus lucasii*, dispersal, philopatry, microhabitat, endemic

## Abstract

The Algerian Cubtail (*Gomphus lucasii* Selys) (Odonata: Gomphidae) is a river-dwelling dragonfly and one of the least known gomphid in the Palearctic. A survey of the movement patterns and habitat requirements of adults was conducted in the largest currently known population, located in the Seybouse River (Northeast Algeria). Daily mark-release-resighting surveys along a 2.5 km stretch of the watercourse and within plots in terrestrial habitats were carried out; a total of 1,316 individuals were marked. The resighting rate along the watercourse was 8.13% and did not significantly vary with sex and age. Adult spatial distribution differed according to sex and age. Mature females were significantly further from the water than males. Mature males were observed not only along the watercourse but also far from the water, up to 450 m where reproductive pairs in copula were recorded. Preferred maturation and foraging sites were open grasslands and dense wheat fields. Philopatry to reproductive sites had a mean of 1.11%, while philopatry to emergence site was lower (0.4%) and noted only in males. The mean distance of natal dispersal (from emergence to reproductive areas) was 596.5 ± 4.94 m. The mean dispersal distance from one reproductive site to another was 180.97 ± 238.54 m. Both mature males and females preferred fast flowing water, but females were observed to oviposit in relatively small watercourses.

In insects and other animals, displacements occur as a life history trait or as a result of disturbance related to habitat degradation. In both cases, such movements could lead to “dispersal,” i.e., the displacement of individuals away from their natal site (Ricklefs and Miller 1999). Several advantages of such movements have been noted: inbreeding avoidance, potential better habitat quality, lower population density, and escaping natural enemies like predators, parasites, and pathogens (Stenseth and Lidicker 1992). When dispersal is followed by reproduction of individuals, a number of genes are transferred from one population to another (so-called gene flow) which can influence population genetics (Broquet and Petit 2009), avoid local extinctions (Harrison 1991, Holt 1993), and expand the geographic range (Ward and Mill 2007, Angert et al. 2011) of a species in a given area.

Odonates have often been used in mark-recapture studies to assess dispersal and demographic parameters (Cordero-Rivera and Stoks 2008). The general movement pattern of odonates during the flight season is that both sexes leave the water after emergence, spend a period of time in terrestrial habitats to mature then return to the water to reproduce. Mature males spend most of their daytime near the water while females visit the water only to oviposit then go back to terrestrial areas (Corbet 1999, Stoks and Córdoba-Aguilar 2012). As a result, a marked male bias in recapture rates within reproductive sites is usually observed. For example, the mean recapture rate based on empirical data of some odonates (expressed as male/female) was estimated to 0.266/0.152 for Coenagrionidae, 0.317/0.119 for Lestidae, and 0.727/0.200 for Libellulidae (Stoks 2001, Cordero-Rivera and Stoks 2008). Some of the scarce data available on dragonfly movements have shown that some species, especially of temporary water, exist in patchy population or metapopulation structure, dispersing substantial distances from one population to another with subsequent gene transfers (Knaus and Wildermuth 2002, Chin and Taylor 2009).

The Algerian Clubtail (*Gomphus lucasii* Selys) (Odonata: Gomphidae) is currently listed vulnerable in the IUCN Red list and is probably one of the least known gomphids in the Palearctic. Its geographic range is restricted to Algeria and Tunisia where most populations have either become extinct or on the verge of extinction during the last century due to anthropogenic pressure (Boudot 2010). It has been claimed that no population currently reaches 250 adults and the total population size is estimated at less than 2,500 individuals (Boudot 2010, Zebsa et al. 2015).

To determine the population dynamics and establish an effective conservation plan, a good understanding of dispersal capacities is essential for species of conservation concern like *G. lucasii* Selys. In addition, data on habitat preferences of maturation and reproductive sites are also crucial because habitat physical characteristics influence dispersal decisions of individuals. Unfortunately, nothing of the biology and ecology of *G. lucasii* Selys has been reported (but see Hamzaoui et al. 2015). In this article, we first investigated adult displacements within and between aquatic and terrestrial habitats using mark-release-resighting data from a large population to determine potential areas occupied by the species during the flight season, its dispersal capacities, and philopatry to emergence and reproductive sites. In addition, microhabitat preferences of reproductive adults were surveyed to investigate physical characteristics of areas where territoriality, copulation, and oviposition often take place.

## Materials and Methods

### 

#### Study Sites

**Fig. 1.**
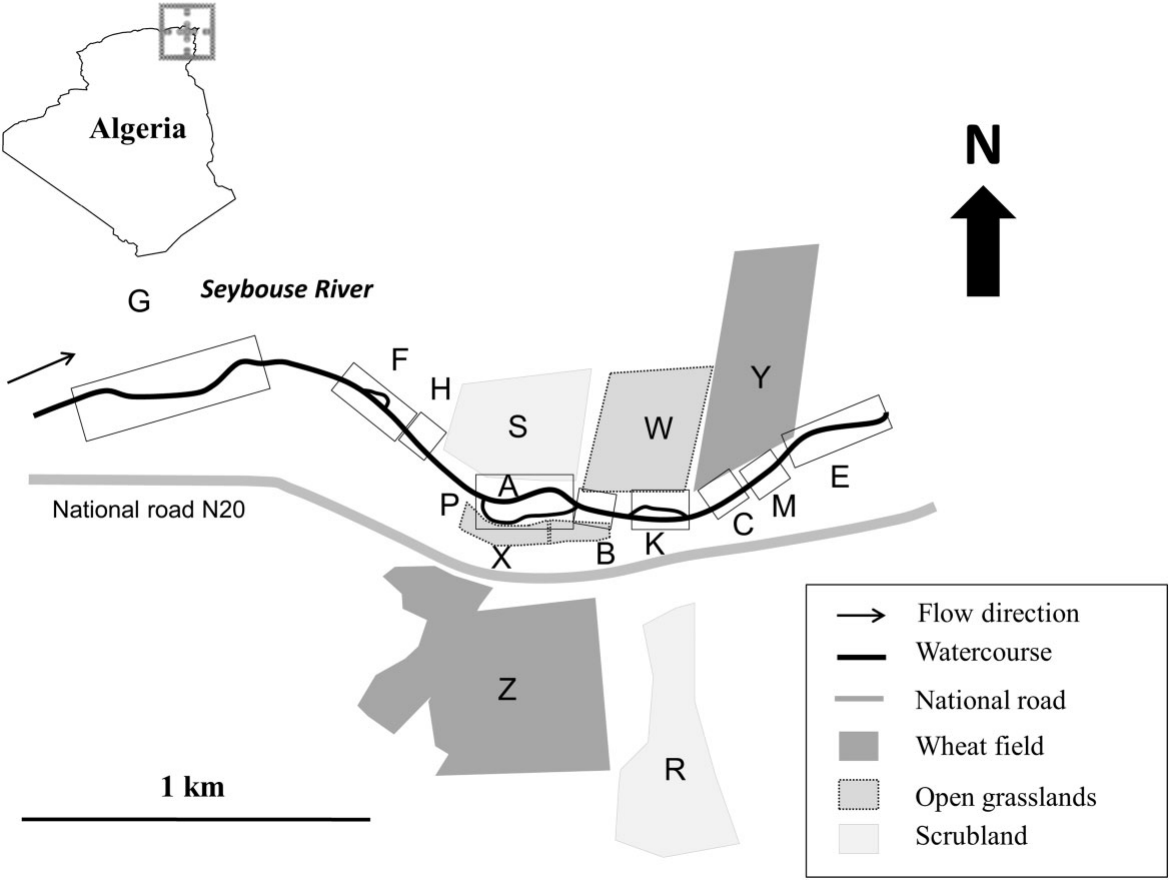
Geographic location of the study site with all habitats and watercourse sections sampled. The following codes: A, B, C, E, F, G, H, K, M, and P represent the sampling watercourse sections. P is the branch (channel) of the watercourse that is parallel to A. R, S, W, X, Y, and Z represent the sampling plots in terrestrial habitats.

This study was carried out on the upstream part of the Seybouse River situated in northeast Algeria, 5 km west from Guelma city (36° 28′ N, 7° 22′ E) (Fig. 1). This part of the river is known to harbor odonates of conservation concern (Khelifa et al. 2012, Khelifa 2013). We limited the study site to a 2.5 km stretch of watercourse with bank vegetation mainly consisting of *Typha angustifolia *L., *Cyperus longus *L., *Tamarix gallica *L., and *Paspalum distichum *L. Away from the watercourse, two plots (2 ha each) in each of the following habitats were surveyed: scrubland of *Olea europea *L. (S and R), open grasslands with sparse *Carduus* *defloratus *L. (W and X), and dense wheat fields dominated by *Triticum aestivum *L. (Y and Z). The odonate community observed along the watercourse consisted of *G. lucasii *Selys, *Onychogomphus costae *Selys, *Orthetrum chrysostigma *Burmeister, *O. coerulescnens *Fabricius, *Calopteryx exul *Sélys, *Calopteryx haemorrhoidalis *Vander Linden, *Ischnura graellsii *Rambur, *Platycnemis subdilatata *Selys, *Erythromma lindenii *Selys, and *Coenagrion caerulescens *Fonscolombe*.*

#### Mark-Release-Resighting Survey

We divided the 2.5 km stretch of the watercourse into nine sections (Fig. 1, Table 1) usually separated by areas where sampling was difficult due to dense arboreal bank vegetation. In each section, small flags were set every 10 m as labels to localize marked and resighted individuals to the nearest meter (by estimation) during our mark-release-resighting (MRR) surveys. We started our daily 4.5 h MRR at 10:00 on 28-IV-2011 (at the beginning of emergence season) until 08-VI-2011 (when no adult was still observed) along all sections by slowly walking along a 5 m belt near the watercourse with a constant speed. Permanent Edding 780 paint markers (edding AG, Bookkoppel 7, D - 22926 Ahrensburg, Germany) were used to mark adults with individual alphanumeric codes on one of the posterior wings after being captured with a hand net of 40 cm diameter. Early emerging tenerals were kept in a cage for 2–3 h before marking to avoid damages. Thirty-minute MRR surveys along parallel transects were conducted daily in olive scrublands (S and R), open grasslands (W and X), and dense wheat fields (Y and Z). During MRR, individual code, sex, age, location, geographic coordinates collected with a GPS, and behavior (reproductive/nonreproductive) of individuals were noted. Coloration (thorax, abdomen, and legs) and behavior (territoriality, copulation, and oviposition) were used to estimate the age of individuals following three categories: teneral (T), immature (IM), and mature (M) (R.K. unpublished data). Distance from the water was estimated to the nearest meter, visually when possible (<20 m) and using a registered map by drawing a line between the point of marking or resighting and the nearest point of the watercourse using Mapinfo 8.5. The distance between the location of marking and resighting or two subsequent resightings were calculated in the same way. Natal dispersal was considered as the displacement of individuals from emergence to reproduction sites. Philopatry to emergence or reproductive sites was noted when the distance travelled within two subsequent resightings was less than 10 m.

**Table 1.** Physical characteristics of the nine sampling sections of the watercourse

Section	Length (m)	Water width (m)	Water depth (cm)	Water velocity (m.s^-1^)	Bank vegetation height (cm)	Bank vegetation density (%)	Emergent stone density (%)	*N*
A	120	13.50 ± 3.37	102.92 ± 38.28	1.68 ± 0.36	173.75 ± 69.19	53.33 ± 26.31	3.75 ± 8.82	10
B	85	10.56 ± 3.32	84.44 ± 20.07	1.43 ± 0.40	180.56 ± 54.40	57.22 ± 19.22	0	10
C	130	15.40 ± 0.97	116.5 ± 25.17	0.29 ± 0.12	181.00 ± 14.49	67.50 ± 14.58	0	10
E	180	11.58 ± 2.91	107.5 ± 31.87	0.25 ± 0.11	157.50 ± 12.15	49.58 ± 24.16	1.67 ± 5.77	10
F	150	12 ± 2.53	72 ± 2.53	0.46 ± 0.01	193 ± 9.78	86.66 ± 5.23	0	10
G	140	12.50 ± 1.17	50.42 ± 6.56	1.29 ± 0.11	186.25 ± 69.71	59.58 ± 21.47	8.75 ± 13.51	10
H	90	25.20 ± 4.02	60 ± 11.18	60 ± 11.18	0.55 ± 0.03	108 ± 4.47	66 ± 2.23	10
K	140	10.77 ± 2.07	81.92 ± 21.26	0.97 ± 0.21	141.92 ± 37.05	39.62 ± 13.30	0.77 ± 2.77	10
M	80	15.43 ± 0.79	132.86 ± 10.75	0.21 ± 0.02	170.71 ± 9.32	69.29 ± 7.32	0	10
P	235	2.81 ± 1.50	39.04 ± 23.05	0.40 ± 0.22	217.39 ± 83.31	73.26 ± 16.14	2.39 ± 8.77	10

#### Reproductive Site Preferences

According to our observations on marked mature males of *G.lucasii *Selys, we are confident that the species is territorial because it may stay at the same territory up to 6 h, which corresponds approximately to day time when the species is reproductively active. Thus, males guard territories at the bank vegetation and on emergent stones along the watercourse. Females come to the water only to lay their eggs and go away to terrestrial habitats. To determine habitat requirements of mature adults, a set of habitat parameters was measured in mid-May in all nine sections, namely water width, water depth, water velocity, height and density of bank vegetation, and percent area covered by emergent stones. A 50-m measuring tape was used to measure water width to the nearest meter. Water depth was measured to the nearest centimeter by a graduated stick at the center of the watercourse bed. By timing a floating object over a 10-m stretch of the watercourse with a chronometer, current velocity (m.s^−1^) was estimated. Bank vegetation height and density were estimated by taking the mean of 10 randomly chosen sampling points. The former parameter was measured to the nearest cm using 5 m measuring tape while the latter was estimated with a 1 by 1 m quadrate to the nearest 5%. Percent area covered by emergent stones was estimated in 10 sampling points randomly chosen in the waterbed using 1 by 1 m quadrate.

#### Statistical Analyses

Most statistical analyses were computed using SPSS 17.0 (SPSS Inc. 2008, SPSS Statistics for Windows, Chicago, IL). When residuals were not normally distributed, nonparametric tests were conducted. In the analysis of distances travelled by the species, age was not included because the time between two consecutive resightings may last a few days, and thus the age class from one observation to the other may change. Mann–Whitney and Kruskal–Wallis tests were used to assess differences in the distance travelled from emergence to maturation sites, the number of marked individuals in each terrestrial habitats, and adult distribution along the watercourse between sexes and age classes. The post-hoc Nemenyi test was computed for pairwise comparison of age classes, using the R package PMCMR (Pohlert 2015). The number of resightings and the time interval (in days) between two subsequent resightings were considered as count data, and thus a generalized linear model (GLM) was used to determine whether there are significant differences in the latter parameter between sexes with a Poisson and quasi-Poisson distribution, respectively, and a log link function. We used quasi-Poisson in the analysis of time interval between two subsequent resightings to overcome overdispersion. A Chi-square test was computed to search for differences between upstream and downstream movements of mature individuals. Chi-square tests were conducted to test for differences in resighting rate between sexes and ages. Spearman’s correlations were used to calculate relationships between abiotic factors and the number of marked mature adults in all sections. Oriana 4 (Kovach Computing Services, 85 Nant-y-Felin, Pentraeth, Anglesey, Wales, UK) was used to analyze circular data and generate a rose diagram. Circular-linear correlation was performed to test for the relationship between the angle of direction and the distance travelled (Solow et al. 1988). Rao's spacing test (*U*) was used to test uniformity of displacement’s directions from emergence to maturation sites. Spearman’s correlations were carried out between the number of territorial males and the number of ovipositing females captured and resighted during the reproductive season and six different abiotic factors. Values are presented as mean ± SD.

## Results

### 

#### Marking and Resighting

In total, 1,316 individuals (870 males and 446 females) were marked during the whole study period in all habitats. Along the watercourse, 775 individuals (579 males and 196 females; representing 58.89% of all marked adults) were marked. The remaining imagos, i.e., 541(291 males and 250 females), were marked far from the water with 314 (151 males and 163 females) in open grasslands and 227 (140 males and 87 females) in dense wheat fields. Combining all habitats, the sex ratio based on regular marking was male biased with 66.11% (χ^2 ^= 126.39, *P* < 0.0001). It was significantly male biased at the watercourse (74.71%; χ^2 ^= 12.37, *P* < 0.0001) and dense wheat fields (61.67%; χ^2 ^= 189.27; *P* < 0.0001) but not significantly female biased in open grasslands (51.91 %; χ^2 ^= 0.45; *P* = 0.49). The overall resighting rate at the watercourse was 8.13%, and it did not vary according to sex and age (Table 2). Males were slightly but not significantly more often resighted than females among tenerals and mature imagos but not among immatures. The highest resighting rate was noted in mature individuals which was quite similar to that of tenerals and about twice that of immature adults (Table 2).

**Table 2.** Marking, resighting, and resighting rate of *G. lucasii* adult according to sex and age

		Teneral	Immature	Mature	Total	χ^2^ (*P*)
Male	Mark	144	71	364	579	
	Resightings	14	2	33	49	
	Resighting rate (%)	9.72	2.82	9.07	8.46	0.18
Female	Mark	115	26	55	196	
	Resightings	8	2	4	14	
	Resighting rate (%)	6.96	7.69	7.27	7.14	0.99
Total	Mark	259	97	419	775	
	Resightings	22	4	37	63	
	Resighting rate (%)	8.49	4.12	8.83	8.13	0.30
χ^2^ (*P*)		0.19	0.12	0.18	0.21	

Chi-square tests in the lower row test for differences between sexes, while those presented in the last column test for differences between age classes.

There were no significant differences in the number of resightings per lifetime between sexes (Poisson GLM: *z* = 0.32, *P* = 0.74). Males were resighted up to four times (1.32 ± 0.71) and females up to twice (1.21 ± 0.42). The time interval between two subsequent resightings was not significantly different between males (5.24 ± 5.62 d) and females (3.94 ± 4.24 d) (Quasi-Poisson GLM: *z* = 1.39, *P* = 0.16) ranging from 1 to 27 d with a mean of 4.78 ± 5.20 d. The maximum observed lifespan was 27 d for males and 21 d for females.

#### Adult Spatial Distribution

**Fig. 2.**
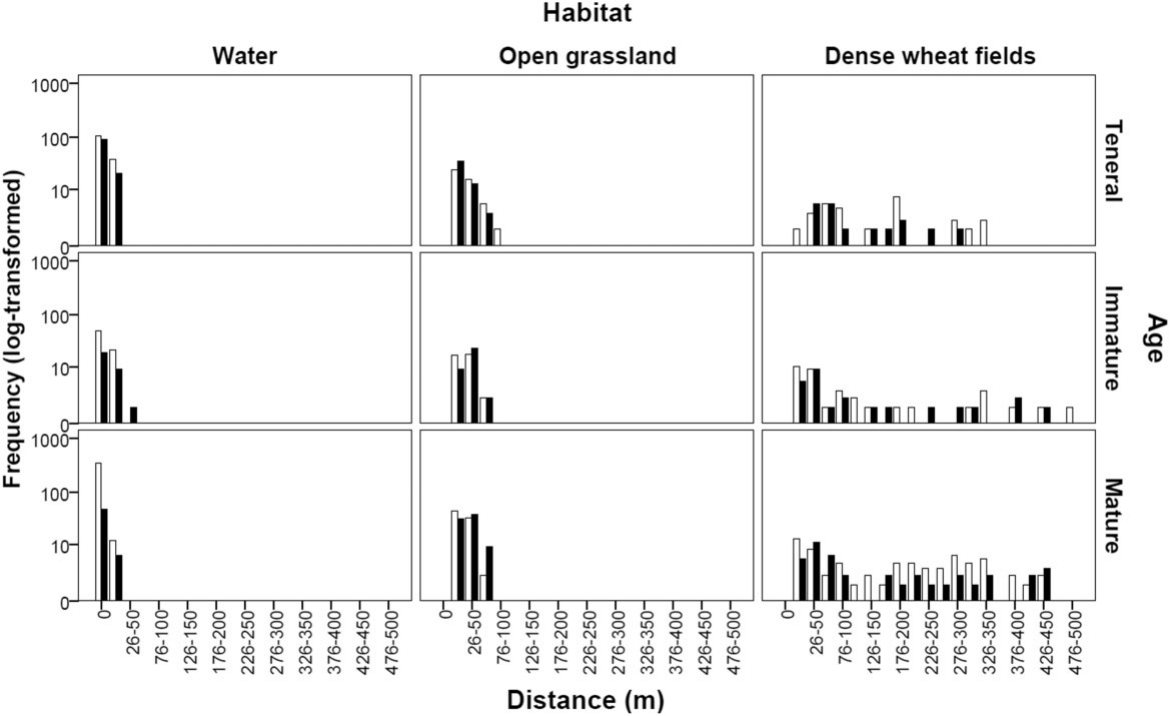
Frequency distribution of distances from the water of *G. lucasii *Selys individuals according to age and habitat types. *Y* axis is in the logarithmic scale. Black bars present males and open bars represent females.

There were significant differences in adult distribution between sexes (Mann Whitney *U*: *P* < 0.0001) and age classes (Kruskal–Wallis: *P* < 0.0001). Pairwise comparisons using Nemenyi test showed that except tenerals and immatures in males (*P* = 0.16), and immatures and matures in females (*P* = 0.47), all age class combinations showed significant difference in distance from the water (*P* < 0.0001). Figure 2 presents the spatial distribution of *G. lucasii* adults for both sexes and three age classes in the three habitat types. Distances from the water of tenerals and immature individuals did not differ significantly between sexes, but mature females were significantly further from the water than males (Table 3). Mature males were mainly recorded at the watercourse (67.84%), but about the third (32.16%) was also recorded at distances ranging from 1 to 450 m from the water. A substantial fraction of mature males (44.73%) recorded between 101 and 450 m were in copula.

**Table 3.** Mean distance from the water of adult individuals of *G. lucasii* for both sexes and three age classes

		*T*	Immature	Mature
Male	Mean	25.87	68.87	25.55
	SD	60.03	113.03	75.06
	Min	0	0	0
	Max	330	500	450
	*N*	216	105	549
Female	Mean	17.13	62.84	28.93
	SD	40.07	96.09	46.18
	Min	0	0	0
	Max	300	450	300
	*N*	183	75	188
Mann–Whitney *U* test (*P*)		0.71	0.05	<0.0001

Away from the watercourse, adults were noted at open grasslands and dense wheat fields but never at shrubby and arboreal maquis. Males had no marked preferences between open grasslands and dense wheat fields (*U* = 874, *P* = 0.23), whereas females preferred open grasslands (*U* = 769, *P* = 0.03).

#### Adult Movements

**Fig. 3.**
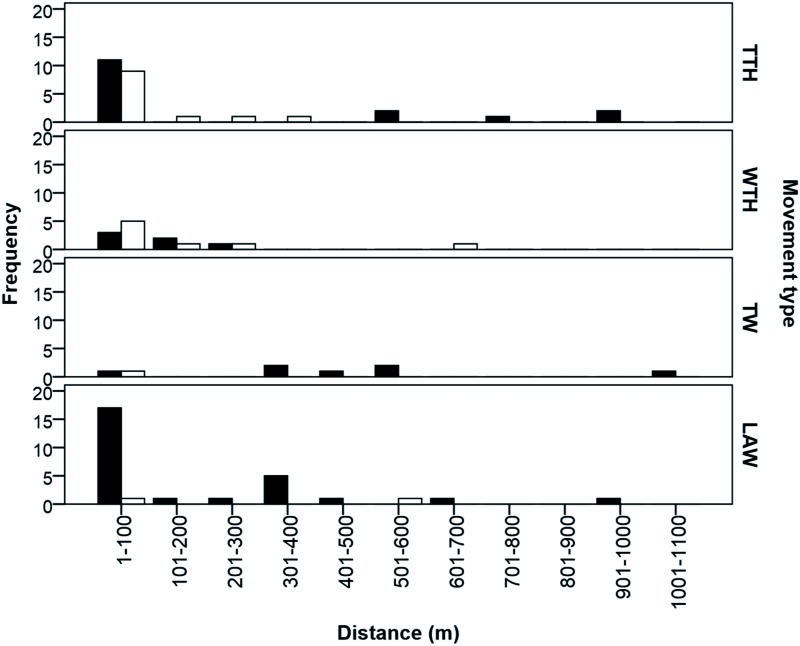
Frequency distribution of distances travelled by *G. lucasii *Selys individuals within and between terrestrial and aquatic habitat. Black bars present males and open bars females present. LAW, lateral along the watercourse; TTH, toward terrestrial habitats; WTH, within terrestrial habitats; TW, toward the water.

**Fig. 4.**
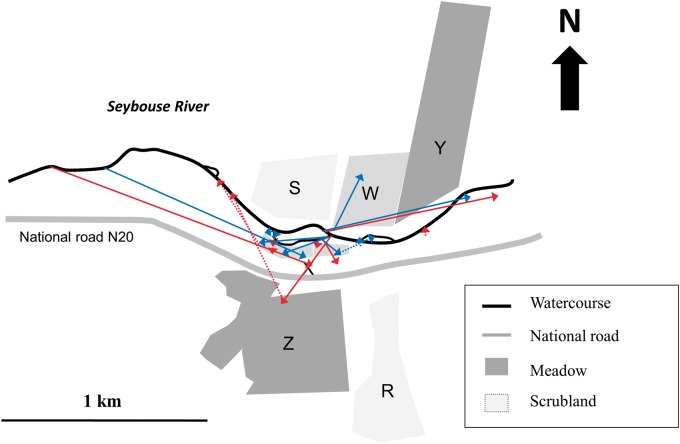
Map showing movements of *G. lucasii *Selys from emergence to maturation sites and from terrestrial habitat to the water. Blue and red color represent females and males, respectively. Continuous and dotted lines refer to movements from emergence to maturation sites and from terrestrial habitat to the water, respectively. Only distances longer greater than 10 m are drawn.

The frequency distribution of the four different kinds of adult movements is presented in Figure 3. From 399 marked tenerals, only 2 were observed at the same site the day after (displacement of <5 m). Only three teneral males (0.75% of tenerals and 1.38% of teneral males) were resighted as mature at the watercourse, showing agonistic behavior toward conspecifics, of which one showed philopatry to its emergence site (9 m from his emergence position) and two showed a mean natal dispersal distance of 596.5 ± 4.94 m (*N* = 2). There were no significant differences in the distance travelled from emergence to maturation sites between sexes (*U* = 107, *P* = 0.96) with a mean distance of 253.75 ± 353.23 m (9–354 m; *N* = 18) for males and 95.75 ± 106.79 m (4–980 m; *N* = 12) for females (Fig. 4).

**Fig. 5.**
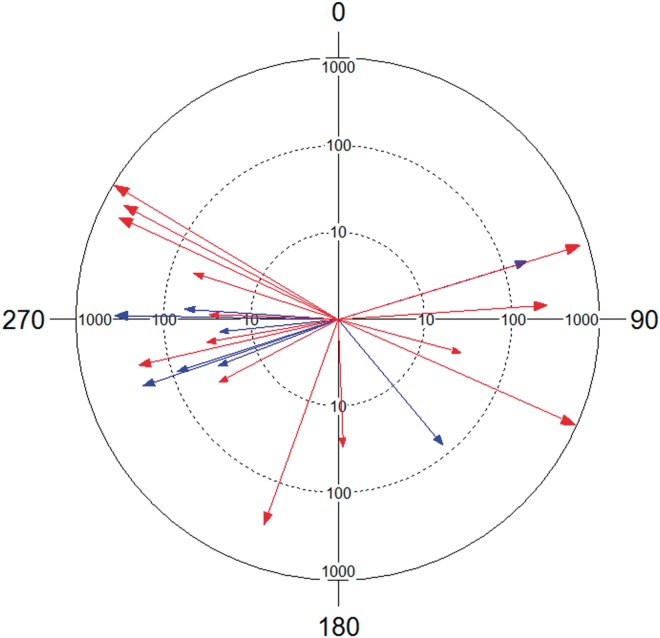
Direction and distance of movements of *G. lucasii *Selys from emergence to maturation sites. Red, male (Rao's Spacing Test (*U*): *r* = 0.29; *U* = 160.857; *P* > 0.05; No. observation = 14; mean vector = 250.84°); blue, female (Rao's Spacing Test (*U*): *r* = 0.56; *U* = 200; *P* < 0.01; No. observation = 8; mean vector = 250.64°). The length of each vector represents the movement distance. Only movements longer than 20 m were considered. Distances are log transformed for display purpose.

When we split data according to sex, a relatively random circular distribution pattern of movement from emergence to maturation sites was noted in males but not in females (Fig. 5). In males, 35.71% displaced in directions between 272° and 301° (i.e., north-westward), 28.57% between 200° and 260° (south-westward), 28.57% between 86° and 178° (south-eastward), and 7.14% in an angle of 73° (north-eastward). Females showed, to some extent, similar movement directions to males with 50% moving between 249° and 264° (south-westward), 25% between 271° and 274° (westward), 12.5% at an angle of 140° (south-eastward), and 12.5% along an angle of 73° (north- eastward). Movement directions and distances were positively correlated in males (Circular-linear correlation: *r* = 0.53, *P* = 0.04) but not in females (Circular-linear correlation: *r* = 0.31, *P* = 0.60).

From 451 individuals marked at open grasslands and dense wheat fields, only 11 (10 males and 1 female) were resighted at their reproductive sites, traveling a mean distance of 376.90 ± 278.82 m (Fig. 4), and 14 carried out displacements within terrestrial habitats with a mean distance of 133.57 ± 177.39 m (Table 4).

**Table 4.** Distance of mature adult movements in the sampling area

	TTH		WTH		TW		LAW	
	Male	Female	Male	Female	Male	Female	Male	Female
Mean	253.77	95.75	123.67	141.00	406.00	78.00	175.15	259.50
SD	353.22	106.78	93.72	228.08	274.70	*	235.94	364.16
Min	4	9	23	2	17	*	2	2
Max	980	354	259	681	969	*	969	517
No. observations	18	12	6	8	10	1	27	2

Distance unit is the meter. LAW, lateral along the watercourse; TTH, toward terrestrial habitats; WTH, within terrestrial habitats; TW, toward the water.

*since sample size is 1, SD, min and max are not calculated.

The mean distance of movements carried out along the watercourse by mature adults was 180.97 ± 238.54 m (Table 4). There were no significant differences between upstream and downstream movements (χ^2 ^= 0.61, *P* = 0.43) with 57.69% of displacements conducted upstream and 42.31% carried out downstream. There were no significant differences between the distance traveled upstream and downstream (*U* = 75, *P* = 0.69). The number of cases of philopatry to reproductive sites was 1 (1.88%) and 3 (0.97%) for females and males, respectively, giving a mean of 1.11%. Only one case of philopatry to emergence site (0.4%) was noted. It was a male who returned after 11 d to relatively the same position of his emergence (9 m).

#### Reproductive Site Preferences

Mature adult spatial distribution was approximately homogenous along the different sections of the watercourse for both males (Kruskal–Wallis: *χ*^2 ^= 14.58, *P* = 0.06) and females (*χ*^2 ^= 14.65, *P* = 0.06). The number of territorial males along the watercourse was positively correlated to water velocity (Spearman’s correlation: *r* = 0.35, *P* < 0.0001) but not to other variables (Table 5). The number of ovipositing females recorded was positively correlated to water velocity (Spearman’s correlation: *r* = 0.23, *P* < 0.0001) and negatively related to water width (*r* = −0.22, *P* = 0.01). Habitat characteristics of territorial males and ovipositing females are presented in Table 5.

**Table 5.** Habitat characteristics of territorial males and ovipositing females

	Water width (m)		Water depth (cm)		Water velocity(m.s^−1^)		Bank vegetation height (cm)		Bank vegetation density (%)		Percent area covered by stones (%)	
	Male	Female	Male	Female	Male	Female	Male	Female	Male	Female	Male	Female
Mean	11.71	6.48	78.28	62.50	0.86	1.12	174.15	203.13	62.46	56.88	4.51	10.00
SD	6.02	4.16	39.21	37.61	0.57	0.43	63.67	58.43	22.41	17.31	10.29	18.52
Min	0.7	2.1	15	15	0.2	0.8	15	130	5	25	0	0
Max	27	12	170	110	2	2	350	280	90	80	40	40
No. observations	71	8	71	8	71	8	71	8	71	8	71	8
Spearman’s correlation *r*	−0.01	−0.22	−0.12	−0.11	0.35	0.23	−0.07	0.10	−0.10	−0.13	0.17	0.11
*P*	0.97	0.01	0.18	0.21	0.00	0.01	0.46	0.28	0.27	0.15	0.06	0.24
df	124	124	124	124	124	124	124	124	124	124	124	124

Sections that were not or rarely frequented by mature individuals were not considered in the calculation of the mean and SD. Spearman’s rank correlations were carried out between the abiotic factors and the mean number of individuals captured or resighted during the reproductive season.

## Discussion

This study first suggests that *G. lucasii *Selys has a high dispersal capacity and second highlights the importance of terrestrial habitats not only as maturation or foraging sites but also as potential refuge for copulating individuals. The general movement pattern can be summarized as follows: tenerals took their first flight away from the water a few hours after their emergence, heading to maturation sites including open grasslands and dense wheat fields and returning to the water after their maturation to reproduce. Both sexes preferred fast flowing water during their mature life, but oviposition was conducted mainly in relatively small watercourse.

Considering the number of individuals marked during this study which exceeds a thousand, we prove that the statement made by the IUCN, i.e., no population exceeds 250 individuals in the entire home range of *G. lucasii *Selys and that the global population is about 2,500 individuals (Boudot 2010), is simply false. This has been proved not only using adults but also with an exuviae-based study which recorded approximately 1,250 exuviae within 60 m stretch (Zebsa et al. 2015). We are confident that our study population does not represent half of the global population and that the latter has been underestimated by the IUCN.

The resighting rate at the watercourse was very small (8.13%) with respect to other dragonflies like *Somatochlora alpestris *Selys (10.2%), *Leucorrhinia hudsonica *Selys (31%), and *Orthetrum coerulescens *Selys (40–55%) (Knaus and Wildermuth 2002, Kéry and Juillerat 2004, Chin and Taylor 2009). Moreover, less than 1% of *G. lucasii *Selys tenerals (only males) were resighted as mature at the water and an even smaller proportion was recorded at their emergence site (philopatry). In addition, resighting rate was not significantly different between sexes and age classes. The relatively homogeneous resighting rate between sexes and age classes might be the result of similar mortality and/or dispersal rate; however, the small number of resightings might also play a role. In some large dragonflies at lentic water habitats, higher resighting rates of tenerals were recorded with 4–14% of males resighted in *S. alpestris* Selys (Knaus and Wildermuth 2002) and higher philopatry to emergence sites with 36–50% of males in *Cordulia aenea amurensis* Selys (Ubukata 1981), 14% of males in *C. a. aenea* (Ha 2000), and 8.5% of males and 18.2% of females in *Aeshna cyanea *Müller (Inden-Lohmar 1997). In contrast to our findings, Cook (1991) found that the recapture rate of mature males (61%) of the territorial *Leucorrhinia frigida *Hagen was three times greater than that of tenerals (22%). We are confident that the low resighting rate recorded in our study was not due to a high mortality but rather to a high dispersal probability given the long distances of movements recorded. It is also probable that in general, dragonflies of lentic water show higher philopatry to their reproductive or emergence sites than river-dwelling dragonflies.

Adult distribution differed according to sex and age. Since our sampling was carried out in the morning, teneral distribution could give a good picture on the maiden flight of the species which was oriented out of the water, in different directions, and could reach 330 m away from the watercourse. However, we are not sure whether these maiden flights were conducted by one or several consecutive flights. Smaller dragonflies like *Leucorrhinia dubia *Vander Linden and *Leucorrhinia rubicunda *Linnaeus carried out maiden flights of 50 m (Pajunen 1962, Soeffing 1990). Unlike Byers (1930) who noted that species of *Gomphus* were attracted to trees during their maiden flights, no teneral of *G. lucasii *Selys was observed perched on a tree or in woodland areas. Habitats preferred by tenerals were open grasslands and dense wheat fields in which the species spent its maturation period. We showed that females preferred to spend their maturation period in open grasslands where the sex ratio was slightly female biased, while males chose both dense wheat fields and open grasslands with no preference, even though sex ratio was male biased at dense wheat fields. The southwest European *Gomphus pulchellus *Selys was observed in similar habitats (Robert 1958).

Mature *G. lucasii* females were usually recorded further from the water than males during their mature life. In territorial dragonflies, females usually go to the water only to lay their eggs and return to terrestrial habitats until the next oviposition, while males stay at the water holding territories and wait for females to reproduce (Corbet 1999). A similar pattern was observed in *G. lucasii *Selys,** but mature males were also observed out of the water solitary or in copula, up to 450 m away from the water. It is likely that pair formation occurred at the water and in terrestrial habitats since both mature males and females occurred far from the water. In addition, pairs in copula recorded in terrestrial habitats could be formed at the watercourse and then went further from the water to avoid male harassment which could reduce the survival and reproductive success of individuals (Stoks and Córdoba-Aguilar 2012).

One nonmarked male was observed out of the study site in Guelma city at 2 km away from the nearest watercourse (R.K., personal observation). Such displacements far from the water are known for species of *Gomphus.* For example, the Eurasian *Gomphus vulgatissimus *Linnaeus was noted at 800 m away from the water (Robert 1958), while the North American *Gomphus ventricosus *Walsh was observed 3 km away from the closest larval habitat (Commissioned by the Committee on the Status of Endangered Wildlife in Canada (COSEWIC) 2010). The mean distance of natal dispersal was 596.5 ± 4.94 m (only males). Male territoriality at the water might be the reason of such long dispersal from emergence site (Stettmer 1996, Chaput-Bardy et al. 2010). The mean distance travelled along the watercourse was 180.97 ± 238.54 m (2–969 m), which was smaller than that of *Pachydiplax longipennis *Brauer with 430 m (320–1,910 m) (McCauley 2010) and *Leucorrhinia hudsonica *Selys with 210 ± 21 m and 432 ± 78 m representing different years (Chin and Taylor 2009). Dispersal of *G. lucasii* adults along the watercourse was not significantly biased neither upstream nor downstream, which suggests that there was a homogeneous distribution of good habitats in our study site.

Teneral males and females travelled similar distances to maturation sites, but males chose more various directions than females. This was probably due to the fact that females choose some specific habitat features that are not widespread in the landscape which limits their movement to certain directions. The limited choice of directions selected by females combined with a relatively high variance of distances made the direction-distance correlation not significant. However, the sample size is quite small to draw any conclusions. Similarly, the number of individuals resighted was low to establish conclusive interpretation regarding movements from maturation to reproductive areas and displacements within maturation sites. However, our data suggest that individuals did not choose the nearest reproductive areas to their maturation sites and usually displaced further. Within terrestrial habitats, females carried out longer movements than males, which is consistent to their tendency to wander and forage intensively to support their biological needs (Conrad et al. 2002). However, males could also be not as pressed as females to satisfy their energy needs (e.g., Marden, 1989; Marden and Waage, 1990).

Mature males occupied a variety of habitat types along the water, while female showed a marked preference to lay eggs in fast and small watercourse, and this might be the reason behind the marginally significant homogeneous distribution of mature adults across river sections. Dragonfly females usually lay their eggs in areas where the survival of eggs during and after embryonic development are highest (Corbet 1999). It seems likely that *G. lucasii *Selys eggs require highly oxygenated water which explains the microhabitat choice of ovipositing females. In fact, Siva-Jothy et al. (1995) found that *Calopteryx splendens *Harris, a stream-dwelling damselfly*,* had faster embryonic development and higher hatching success in fast-flowing water than slow-flowing water.

Dispersal and habitat use of *G. lucasii* have never been investigated and posed problems in understanding the ecology and population structure of this threatened species in the past. We conclude from this study that an effective conservation should take into account not only the aquatic ecosystem but also the nearby terrestrial habitats because they represent maturation sites for immature individuals, foraging sites for mature females between two oviposition episodes, and copulation areas for reproductive pairs. It is easily predictable from our results that the species can colonize new sites either by flying along the water or by crossing terrestrial habitats, an ability that is crucial for the survival of populations in a continually degraded environment.
